# Supplementary material for: Dynamic transcriptomic profiles of zebrafish gills in response to zinc supplementation
Source: BMC Genomics. 2010 Oct 11;11:553. doi: 10.1186/1471-2164-11-553 (PMC3091702; doi:10.1186/1471-2164-11-553)
Supplement: Additional file 2 — Interactive Direct Interaction Network representing the molecular interactions between zinc, copper, iron, calcium and proteins encoded by transcripts changed by zinc supplementation. Mini web-site containing index.html and hyperlinked pages in subdirectory describing a Direct Interaction Network automatically generated based on curated interactions contained within the proprietary PathwayArchitect database. Ovals represent proteins and the circles symbolize metal ions. Objects are coloured by their abundance in zebrafish at the time-point they were significantly different from the control is a scale from -4 fold (dark green) to +4 fold (dark red). Where significant differences were found at more than one time-point, the colour overlay shows expression at the first instance. Dark blue squares denote 'binding', and light blue squares 'expression'; green squares stand for 'regulation', green diamonds for 'metabolism', and green circles for 'promoter binding'. Arrow heads indicate directionality of the interaction where annotated. All nodes and edges can be further interrogated by selecting the relative area of the image. [file 1471-2164-11-553-S2.zip › PathwayArchitect Zn xs DIN/534087.html]

# REGULATION:

|  |  |
| --- | --- |
| Type | REGULATION |
| Effect | Positive |


---

|  |  |
| --- | --- |
| Score | 0 |


---

|  |  |
| --- | --- |
| Reference Count | 7 |


---

|  |  |
| --- | --- |
| Mechanism | Unknown |


---

|  |  |
| --- | --- |
| Reference:0 || Sentence | "Paraventricular corticotropin releasing hormone (CRF) IR was negatively correlated with the pituitary ACTH IR, indicating that the increase in ACTH release was produced by an increased release of CRF from the hypothalamus." |
| PMID | 2760606 |
| Year | 1989 |
| Species | Rat |
| Journal | J Neural Transm |
| RefScore | 2 |
| Source | PArchNLP |
  |
|  |  |


---

|  |  |
| --- | --- |
 Reference:1 || Sentence | "Corticotropin releasing hormone (CRH), which activates adenylyl cyclase and stimulates adrenocorticotropic hormone (ACTH) secretion from corticotropes, inhibited IK(IR) by 25% and depolarized the cells by 10.2 +/- 1.0 mV." |
| PMID | 9263909 |
| Year | 1997 |
| Species | Rat |
| Journal | J Physiol |
| RefScore | 0 |
| Source | PArchNLP |
  ||  |  |


---

|  |  |
| --- | --- |
 Reference:2 || Sentence | "Previous studies have shown that plasma ACTH immunoreactivity (ACTH-IR) of African American women, measured after ovine CRH (oCRH) stimulation, is significantly greater than ACTH-IR of Caucasian women, but is not accompanied by greater plasma cortisol concentrations." |
| PMID | 10946862 |
| Year | 2000 |
| Species | Human |
| Journal | J Clin Endocrinol Metab |
| RefScore | 3 |
| Source | PArchNLP |
  ||  |  |


---

|  |  |
| --- | --- |
 Reference:3 || Sentence | "Ovine corticotropin-releasing factor (oCRF) stimulates increased plasma immunoreactive adrenocorticotropin (IR-ACTH) and IR-cortisol at threshold, half-maximal, and maximal doses of 0.01-0.03, 0.3-1, and 3-10 micrograms/kg, respectively." |
| PMID | 2981741 |
| Year | 1985 |
| Species | Human |
| Journal | Fed Proc |
| RefScore | 0 |
| Source | PArchNLP |
  ||  |  |


---

|  |  |
| --- | --- |
 Reference:4 || Sentence | "Dual labeling revealed extensive correspondence of CRF-stimulated Fos-immunoreactivity (Fos-ir) and CRF-R1 mRNA at these and other loci." |
| PMID | 10648719 |
| Year | 2000 |
| Species | Rat |
| Journal | J Neurosci |
| RefScore | 0 |
| Source | PArchNLP |
  ||  |  |


---

|  |  |
| --- | --- |
 Reference:5 || Sentence | "The action of IL-1 beta on ACTH-ir release was annulled by simultaneous exposure to alpha-helical-CRH, an antagonist of CRH." |
| PMID | 7804604 |
| Year | 1994 |
| Species | Rat |
| Journal | Mol Cell Neurosci |
| RefScore | 0 |
| Source | PArchNLP |
  ||  |  |


---

|  |  |
| --- | --- |
 Reference:6 || Sentence | "Such differences in the circulating forms of ACTH can account for greater CRH-stimulated ACTH-IR in blacks." |
| PMID | 8784088 |
| Year | 1996 |
| Species | Human |
| Journal | J Clin Endocrinol Metab |
| RefScore | 0 |
| Source | PArchNLP |
  |


---

|  |  |
| --- | --- |
